# Supplementary material for: Proteomic characterization of the Toxoplasma gondii cytokinesis machinery portrays an expanded hierarchy of its assembly and function
Source: Nat Commun. 2022 Aug 8;13:4644. doi: 10.1038/s41467-022-32151-0 (PMC9360017; doi:10.1038/s41467-022-32151-0)
Supplement: Supplementary file 1 — Supplementary Information [file 41467_2022_32151_MOESM1_ESM.pdf]

Supplementary Information

Proteomic characterization of the *Toxoplasma gondii* cytokinesis machinery portrays an expanded hierarchy of its assembly and function

Klemens Engelberg[1*], Tyler Bechtel[2], Cynthia Michaud[1], Eranthie Weerapana[2], Marc-Jan Gubbels[1*]

[1]Department of Biology, Boston College, Chestnut Hill, Massachusetts, USA
[2]Department of Chemistry, Boston College, Chestnut Hill, Massachusetts, USA

*Corresponding authors: engelbek@bc.edu; gubbelsj@bc.edu

Supplementary Figure 1

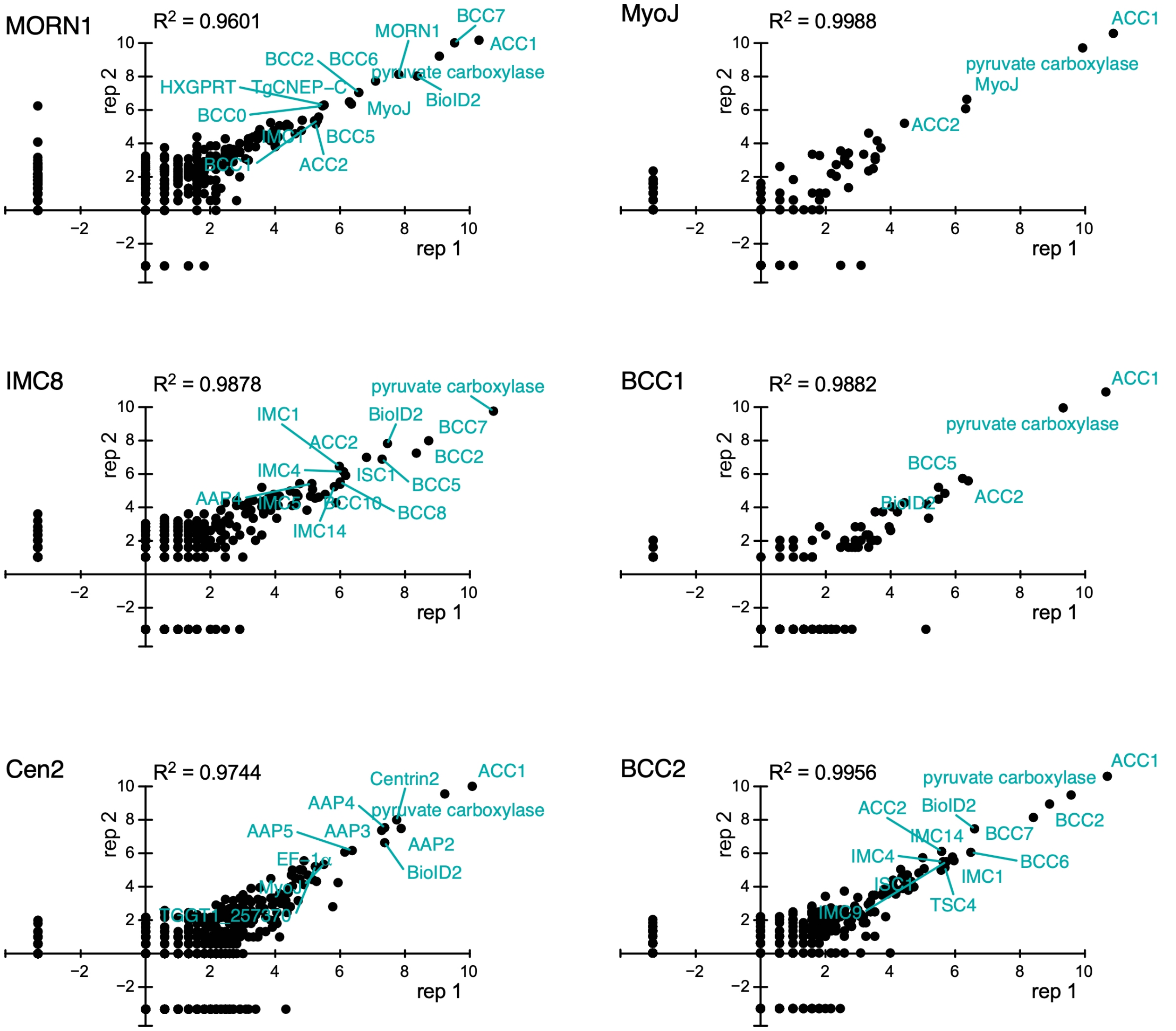

Supplementary Fig. 1: Comparison of two biological replicates for each bait used to generate the Basal Complex BioID data set.

Spectral counts (SpC) for each biological replicate (average of two technical replicates) were log2 transformed and plotted. A pseudo count of 0.1 was assigned to missing preys before log-transformation. Preys that were identified with ≥ 32 SpC (log2 = 5) are highlighted with annotation/geneID (seafoam) in the plot. IDs of BioID baits are indicated in the upper left corner of the respective plots. R^2-values were calculated for biological replicates based on SpC (not log2 transformed) to prevent distortion from the addition of pseudo counts. Source data are provided as a Source data file.

Supplementary Figure 2

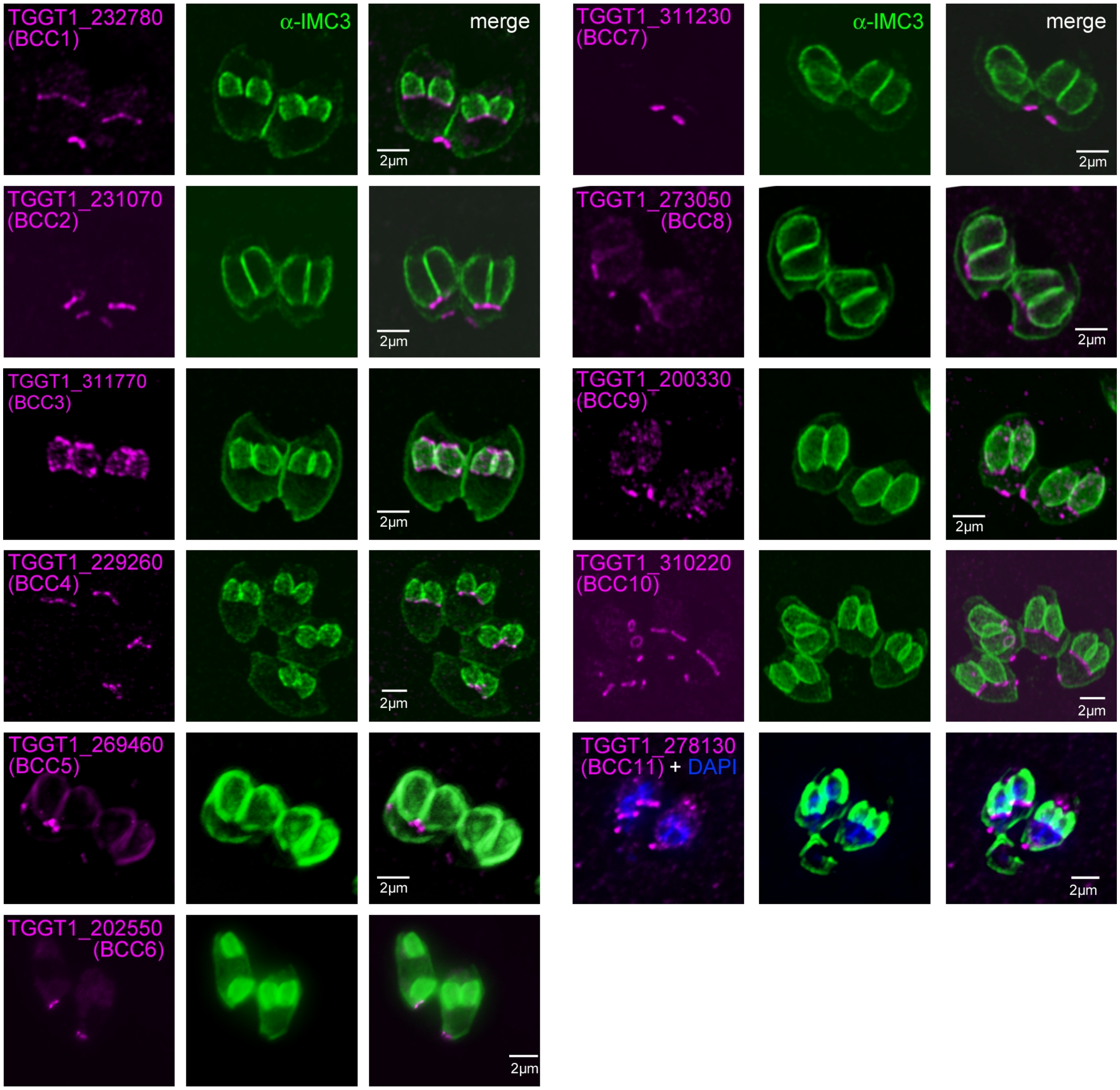

Supplementary Fig. 2: Localization patterns of BCC components. All genes were endogenously tagged and co-stained with IMC3 antiserum. All parasite images are of mid-budding to provide a general overview of the spatio-temporal localization dynamics between mother and daughter parasite BC and/or cytoskeleton.

Supplementary Figure 3

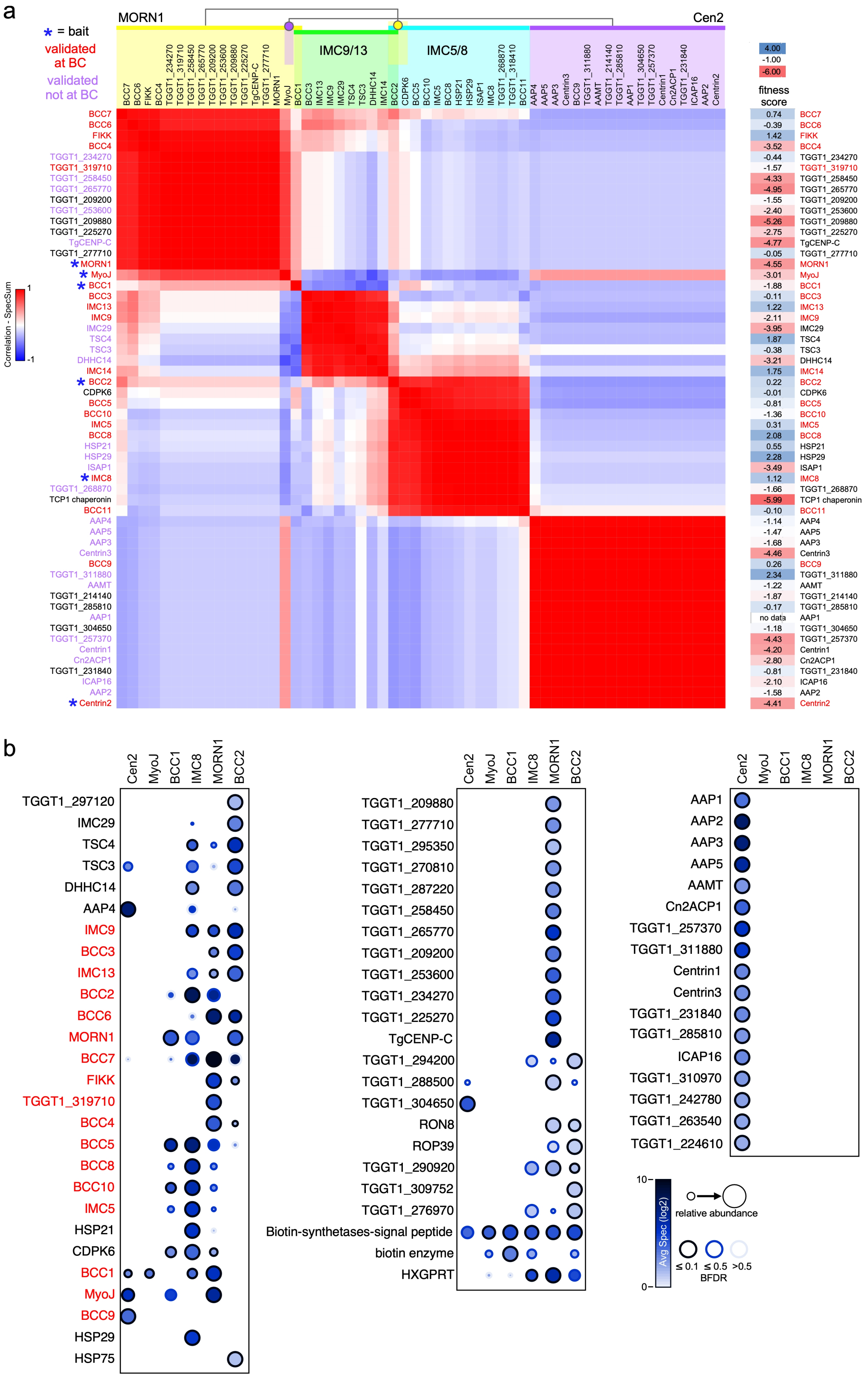

Supplementary Fig. 3: Extended statistical analysis of proximity biotinylation results.

a Prey-prey correlation map including the BioID2 fusion baits and including cytoplasmic YFP-BioID2 as a negative control. Note that inclusion of this control actually loosens the stringency of proteins assigned to the clusters. BCC0 was discovered by the settings shown in Fig. 2a Prohits-viz settings for this correlation map were as follows: Abundance column: “SpecSum”, score column: “False discovery rate (FDR)”, score filter: “0.25”, second score filter: “0.1” and abundance cutoff for prey correlation: “20”. Fitness scores[1] for each gene are included on the right side.

b Dot plot including the cytoplasmic YFP-BioID2 as a negative control provides overview regarding the strength of the hits for bait, as indicated. BCC0 and BCC11 did not meet the criteria used to generate this plot. Gene names in red represent experimentally validated BCC components. Settings used to generate the plot are shown in Fig. 2b. Source data are provided as a Source data file.

Supplementary Figure 4

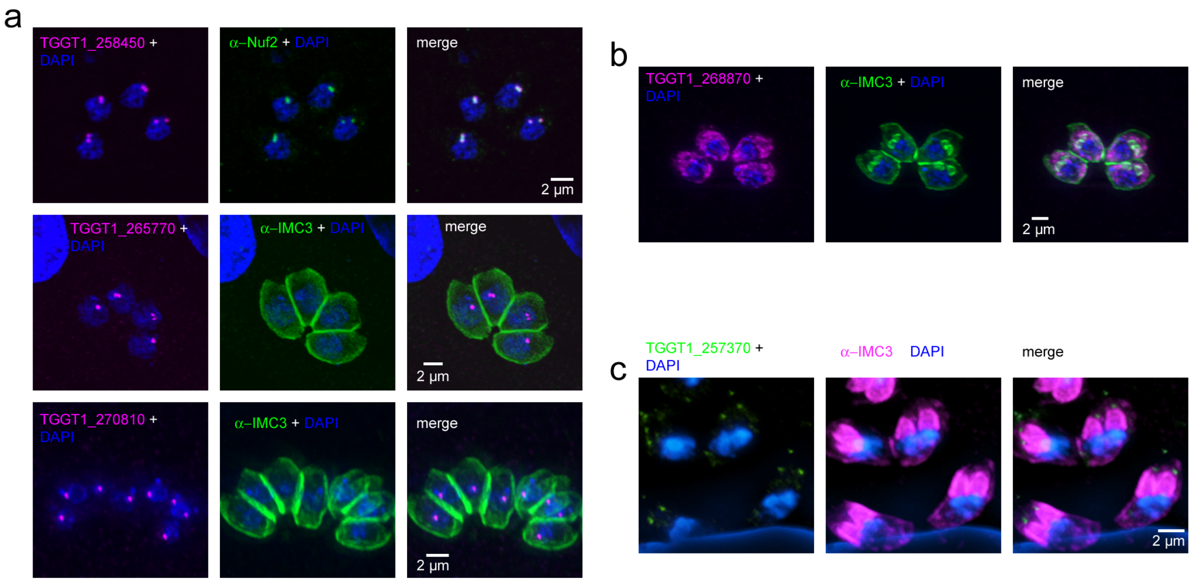

Supplementary Fig. 4: Subcellular localization of proteins unrelated to the basal complex.

a Genes were either tagged at the endogenous 5' (TGGT1_258450 or TGGT1_265770) or 3' end (and TGGT1_270810) with Myc_3 and co-staining with the kinetochore marker Nuf2 or the cytoskeleton marker IMC3. All three proteins showed close association with the nucleus or partial colocalization with Nuf2 (TGGT1_258450) indicating localization to the centrocone.

b Endogenous 3'end tagging of TGGT1_268870 with Myc_3 revealed cytosolic localization for the protein.

c Promoter replacement of TGGT1_257370 with tetO7sag4-Ty and subsequent Ty-staining indicated apical localization for the protein in mother and daughter parasites.

Supplementary Figure 5

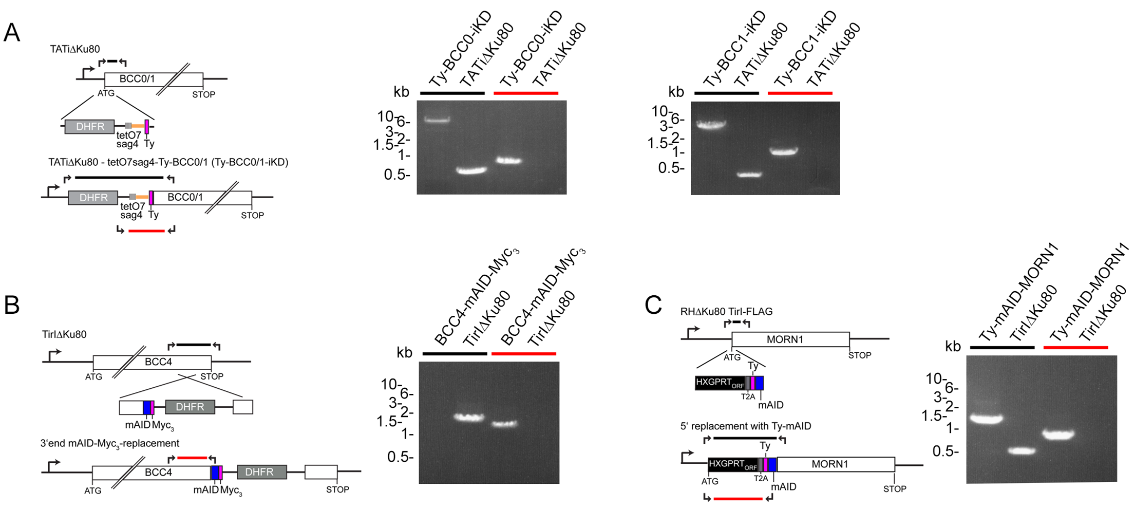

Supplementary Fig. 5: Generation of knockdown/degradation strains.

a Promoter replacement of BCC0 was achieved by a CRISPR/Cas9-induced DNA double-strand break close to the start codon and co-transfection of a PCR amplicon harboring the DHFR selection cassette as well as the regulatable sag4 promoter and a Ty-epitope tag. The amplicon had 35 bp long homologous flanks on each side.

b Endogenous 3’ end replacement of the BCC4 ORF was achieved by transfection of a linearized plasmid containing a large homologous sequence, fused to the mAID and Myc_3-epitope tag sequence. The DHFR cassette was used for selection.

c MORN1 degradation was achieved by replacing its endogenous 5’ end close to the start codon with PCR amplicon containing a Ty-epitope tag and the mAID sequence. The DNA double-strand break was induced by CRISPR/Cas9. The HXGPRT ORF, used for selection, will separate from the Ty-mAID-MORN1 fusion protein at the ribosome due to the T2A skip peptide. The PCR amplicon has 35 bp long homologous flanks on each side.

d The strategy to replace the BCC1 promoter was the same as used for BCC0 in **a.**

Arrows indicate locations of utilized PCR oligomers in all diagrams; black oligomer sets bind in endogenous DNA sections, red combine one endogenous oligomer with one specific to the integrated sequence.

Supplementary Figure 6

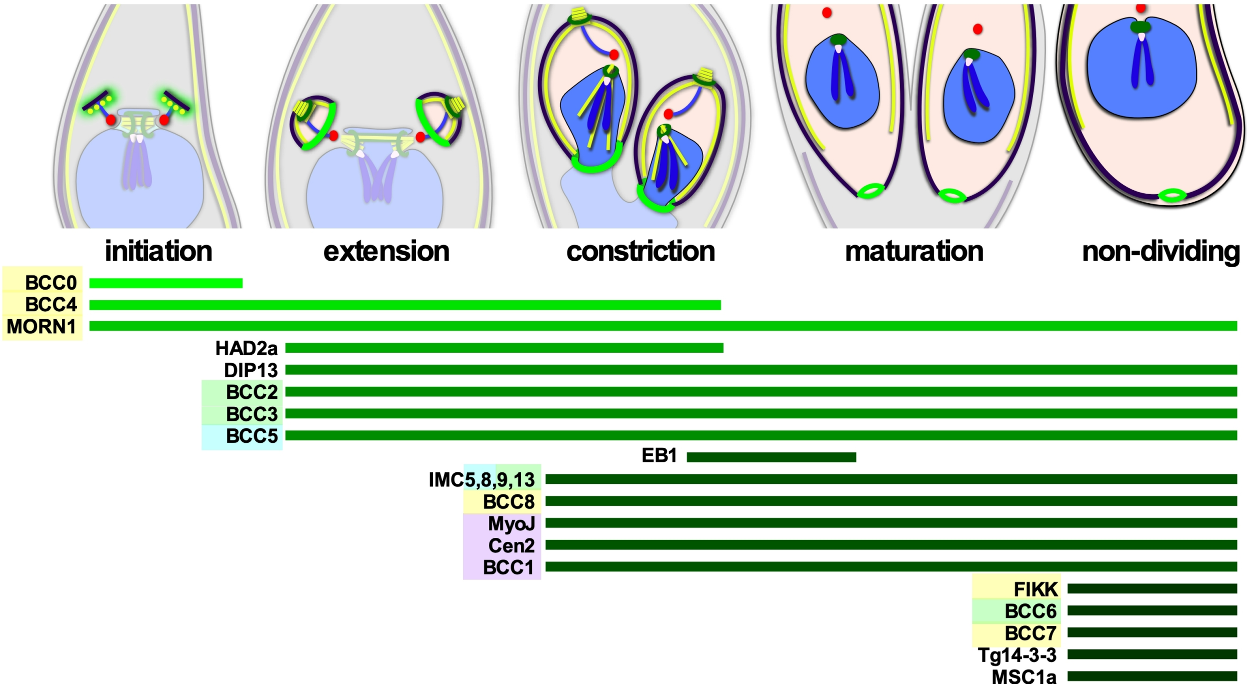

Supplementary Fig. 6: Temporal patterns of BC composition changes coincide with developing BC function. Note that BCSCs are largely not assembled sequentially but predominantly in parallel. Colored blocks marking the protein names correspond with the BCSC complexes as follows: yellow, BCSC-1; green, BCSC-2; bleu, BCSC-2; purple BCSC-4. Original data gathered for: HAD2a[2], DIP13/SSNA1[3], EB1[4], IMC5, 8, 9, 13[5], FIKK[6], and finally Tg14-3-3 and MSC1a[7].

Supplementary Figure 7

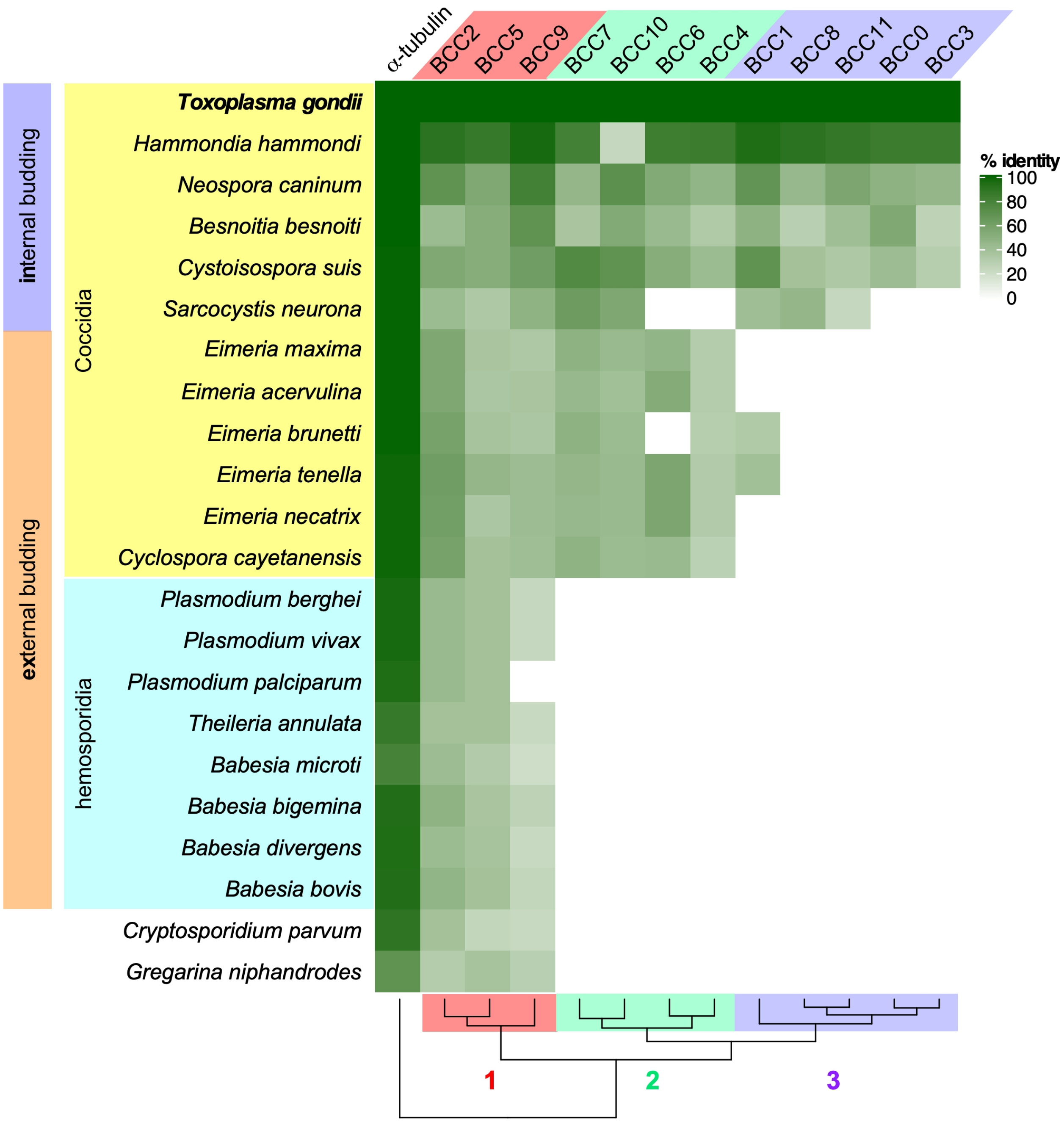

Supplementary Fig. 7: Gene conservation of newly identified BCCs. *T. gondii* BCC protein sequences were BLASTP searched for orthologs on EuPathDB[8] against the following species and strains: *Cryptosporidium parvum* Iowa II, *Cryptosporidium muris* RN66, *Cystoisospora suis* strain Wien I, *Cyclospora cayetanensis* isolate

NF1_C8, *Babesia bovis* T2Bo, *Babesia bigemina* strain BOND, *Babesia divergens* strain 1802A, *Babesia microti* strain RI, *Gregarina niphandrodes* Unknown strain, *Theileria annulata* strain Ankara, *Plasmodium falciparum* 3D7, *Plasmodium berghei* ANKA, *Plasmodium vivax* P01, *Toxoplasma gondii* GT1, *Hammondia hammondi* strain H.H.34, *Neospora caninum* Liverpool, *Sarcocystis neurona* SN3, *Eimeria tenella* strain Houghton, *Eimeria maxima* Weybridge, *Eimeria brunetti* Houghton, *Eimeria acervulina* Houghton. Top hits in each species were selected as orthologs. The percentage identity for the orthologs was used to assemble a heatmap generated in R using the

ComplexHeatmap package[9]. *T. gondii* α-tubulin was used as a reference for a highly conserved gene. Source data are provided as a Source data file.

Supplementary References:

1. Sidik, S. M. *et al.* A Genome-wide CRISPR Screen in Toxoplasma Identifies Essential Apicomplexan Genes. *Cell* 1–26 (2016). doi:10.1016/j.cell.2016.08.019
2. Engelberg, K. *et al.* A MORN1-associated HAD phosphatase in the basal complex is essential for *Toxoplasma gondii* daughter budding. *Cellular Microbiology* **18,** 1153–1171 (2016).
3. Lévêque, M. F., Berry, L. & Besteiro, S. An evolutionarily conserved SSNA1/DIP13 homologue is a component of both basal and apical complexes of Toxoplasma gondii. *Sci. Rep.* 1–14 (2016). doi:10.1038/srep27809
4. Chen, C.-T. *et al.* Compartmentalized Toxoplasma EB1 bundles spindle microtubules to secure accurate chromosome segregation. *Mol. Biol. Cell* **26,** 4562–4576 (2015).
5. Anderson-White, B. R. *et al.* A family of intermediate filament-like proteins is sequentially assembled into the cytoskeleton of Toxoplasma gondii. *Cellular Microbiology* **13,** 18–31 (2011).
6. Skariah, S. *et al.* The FIKK kinase of Toxoplasma gondii is not essential for the parasite's lytic cycle. *International Journal for Parasitology* **46,** 323–332 (2016).
7. Lorestani, A. *et al.* Targeted proteomic dissection of Toxoplasmacytoskeleton sub-compartments using MORN1. *Cytoskeleton* **69,** 1069–1085 (2012).
8. Warrenfeltz, S. *et al.* EuPathDB: The Eukaryotic Pathogen Genomics Database Resource. *Methods Mol. Biol.* **1757,** 69–113 (2018).
9. Gu, Z., Eils, R. & Schlesner, M. Complex heatmaps reveal patterns and correlations in multidimensional genomic data. *Bioinformatics* **32,** 2847–2849 (2016).
