# Supplementary material for: Proteomic characterization of the Toxoplasma gondii cytokinesis machinery portrays an expanded hierarchy of its assembly and function
Source: Nat Commun. 2022 Aug 8;13:4644. doi: 10.1038/s41467-022-32151-0 (PMC9360017; doi:10.1038/s41467-022-32151-0)
Supplement: Supplementary file 3 — Description of Additional Supplementary Files [file 41467_2022_32151_MOESM3_ESM.docx]

Proteomic characterization of the *Toxoplasma gondii* cytokinesis machinery portrays an expanded hierarchy of its assembly and function

Klemens Engelberg^1*^, Tyler Bechtel^2^, Cynthia Michaud^1^, Eranthie Weerapana^2^,

Marc-Jan Gubbels^1*^

^1^Department of Biology, Boston College, Chestnut Hill, Massachusetts, USA

^2^Department of Chemistry, Boston College, Chestnut Hill, Massachusetts, USA

*Corresponding authors: engelbek@bc.edu; gubbelsj@bc.edu

File Name: Supplementary Data 1

Description: Details on the BioID candidates in the prey-prey heatmap of Fig S3.

File Name: Supplementary Data 2

Description: Oligomers and synthetic DNA used in this study.

File Name: Supplementary Data 3

Description: Detected preys over all BioID experiments. Values indicate spectral counts for unique proteins.

File Name: Supplementary Data 4

Description: SAINTexpress readout with or without YFP as cytosolic control. Table show: Bait, prey GeneID (Prey), ToxoDB annotation (PreyGene), detected spectral counts for unique proteins in technical replicates (Spec), Sum of spectral counts (SpecSum), average spectral counts over technical replicates (AvgSpec), number of replicates (NumReplicates), spectral counts for the respective prey in control samples (ctrlCounts), average probability score (AvgP), maximum probability score (MaxP), topology-assisted probability score (TopoAvg), maximum topology-assisted probability score (TopoMaxP), larger of AvgP and TopoAvgP (SaintScore), AvgSpec divided by average spectral counts detected in control samples (FoldChange), Bayesian false discovery rate (BFDR).

File Name: Supplementary Movie 1

Description: SR-SIM 3D rotation of BCC0-smMyc co-expressing endogenously tagged YFP-MORN1 corresponding with Fig. 3d.

File Name: Supplementary Movie 2 and 3

Description: Time-lapse microscopy of BCC4-mAID-Myc_3_ parasites expressing endogenously YFP-tagged MORN1, without (Movie S2) and with IAA (Movie S3). IAA was added at t = - 2 hrs. These movies correspond with Fig. 7d. Parasites were imaged in three minute intervals.

File Name: Supplementary Movie 4 and 5

Description: U-ExM of BCC4-mAID-3xMyc parasites endogenously expressing YFP-MORN1 co-stained with α-tubulin (12G10) antiserum. Movie S4 represents the uninduced wild type, whereas Movie S5 was depleted of BCC4. These movies correspond with Fig. 7e.
