# Supplementary material for: Proteomic characterization of the Toxoplasma gondii cytokinesis machinery portrays an expanded hierarchy of its assembly and function
Source: Nat Commun. 2022 Aug 8;13:4644. doi: 10.1038/s41467-022-32151-0 (PMC9360017; doi:10.1038/s41467-022-32151-0)
Supplement: Supplementary file 13 — Reporting Summary [file 41467_2022_32151_MOESM13_ESM.pdf]

nature portfolio

Corresponding author(s): Klemens Engelberg and Marc-Jan Gubbels

Last updated by author(s): Jul 15, 2022

Reporting Summary

Nature Portfolio wishes to improve the reproducibility of the work that we publish. This form provides structure for consistency and transparency in reporting. For further information on Nature Portfolio policies, see our Editorial Policies and the Editorial Policy Checklist.

Statistics

For all statistical analyses, confirm that the following items are present in the figure legend, table legend, main text, or Methods section.

n/a	Confirmed	
☐	☒	The exact sample size (*n*) for each experimental group/condition, given as a discrete number and unit of measurement
☐	☒	A statement on whether measurements were taken from distinct samples or whether the same sample was measured repeatedly
☐	☒	The statistical test(s) used AND whether they are one- or two-sided<br>*Only common tests should be described solely by name; describe more complex techniques in the Methods section.*
☒	☐	A description of all covariates tested
☐	☒	A description of any assumptions or corrections, such as tests of normality and adjustment for multiple comparisons
☐	☒	A full description of the statistical parameters including central tendency (e.g. means) or other basic estimates (e.g. regression coefficient) AND variation (e.g. standard deviation) or associated estimates of uncertainty (e.g. confidence intervals)
☐	☒	For null hypothesis testing, the test statistic (e.g. *F*, *t*, *r*) with confidence intervals, effect sizes, degrees of freedom and *P* value noted<br>*Give P values as exact values whenever suitable.*
☒	☐	For Bayesian analysis, information on the choice of priors and Markov chain Monte Carlo settings
☒	☐	For hierarchical and complex designs, identification of the appropriate level for tests and full reporting of outcomes
☒	☐	Estimates of effect sizes (e.g. Cohen's *d*, Pearson's *r*), indicating how they were calculated

Our web collection on statistics for biologists contains articles on many of the points above.

Software and code

Policy information about availability of computer code

Data collection	Imaging:<br>Zen 2.3 (black edition)<br>DeltaVision AcquireUltra-1.2.3<br>Volocity6.3
Data analysis	Mass spectrometry:<br>RawConverter 1.1.0.20<br>SEQUEST v3.0<br>DTASelect v2.0<br>SAINTexpress_v3.6.3, ProHits-viz1.0<br>Microsoft Excel:mac2011<br>Imaging:<br>Zen 2.3 (blue edition), FIJI (version 2.3.0/1.53f),<br>Statistics:<br>GraphPad Prism v6.0h, R statistical software v3.6.3, R package ggplot2(ggplot2, version 3.3.3), R package rstatix (rstatix, version 0.7.0),<br>Gene conservation:<br>R package ComplexHeatmaps (ComplexHeatmaps, version 2.2.0)

For manuscripts utilizing custom algorithms or software that are central to the research but not yet described in published literature, software must be made available to editors and reviewers. We strongly encourage code deposition in a community repository (e.g. GitHub). See the Nature Portfolio guidelines for submitting code & software for further information.

Data

Policy information about availability of data

All manuscripts must include a data availability statement. This statement should provide the following information, where applicable:

- Accession codes, unique identifiers, or web links for publicly available datasets
- A description of any restrictions on data availability
- For clinical datasets or third party data, please ensure that the statement adheres to our policy

All relevant data are included in the main text of the manuscript and the supplementary material. Raw data points underlying graphs are present in the Source data file accompanying the manuscript. All mass spectrometry raw data have been deposited to theProteomeXchnage Consortium via the Pride partner repository under the accession code: PXD031116 [https://www.ebi.ac.uk/pride/archive/projects/PXD031116]. T. gondii genomic information can be found in ToxoDB [https://toxodb.org]

Field-specific reporting

Please select the one below that is the best fit for your research. If you are not sure, read the appropriate sections before making your selection.

- [x] Life sciences
- [ ] Behavioural & social sciences
- [ ] Ecological, evolutionary & environmental sciences

For a reference copy of the document with all sections, see nature.com/documents/nr-reporting-summary-flat.pdf

Life sciences study design

All studies must disclose on these points even when the disclosure is negative.

Sample size	All experiments were performed with a large enough sample size to allow statistical analyses (n=3 biological replicates) unless stated otherwise. Sample size was based on previously published studies. Basal diameter measurements were based on (1),sample size for BioID experiments were done similar to (2),:<br>1)Frénal K, Jacot D, Hammoudi PM, Graindorge A, Maco B, Soldati-Favre D. (2017)<br>Myosin-dependent cell-cell communication controls synchronicity of division in acute and chronic stages of Toxoplasma gondii. Nat Commun. 8:1570. PMID: 28593938<br>2) Chen AL, Moon AS, Bell HN, Huang AS, Vashisht AA, Toh JY, Lin AHL, Nadipuram AM, Kim EW, Choi CP, Wohlschlegel JA, Bradley PJ. (2017) Novel insights into the composition and function of the Toxoplasma IMC sutures. Cell Microbiol. 19(4): PMID:27696623
Data exclusions	The authors declare that no data were excluded.
Replication	Experiments were executed as biological replicates, measuring the same clonal line on different days. Unless stated otherwise, three biological replicates were used. All microscopy images are representatives of at least two independent experiments. BioID experiments were done as two biological replicates, which were split into technical replicates for the MS run. All experiments resulted in comparable results.
Randomization	Randomization did not apply. Experiments were conducted by treating samples and controls side by side under the indicated conditions.
Blinding	For measurement of double headed parasites sample identity was hidden and only revealed after analysis was concluded. For other experiments, investigators were not blinded. These experiments reached quantitative endpoints, which are less likely to be influenced by investigator bias.

Reporting for specific materials, systems and methods

We require information from authors about some types of materials, experimental systems and methods used in many studies. Here, indicate whether each material, system or method listed is relevant to your study. If you are not sure if a list item applies to your research, read the appropriate section before selecting a response.

Materials & experimental systems

n/a	Involved in the study	
☐	☒	Antibodies
☐	☒	Eukaryotic cell lines
☒	☐	Palaeontology and archaeology
☒	☐	Animals and other organisms
☒	☐	Human research participants
☒	☐	Clinical data
☒	☐	Dual use research of concern

Methods

n/a	Involved in the study	
☒	☐	ChIP-seq
☒	☐	Flow cytometry
☒	☐	MRI-based neuroimaging

Antibodies

Antibodies used	Primary antibodies:<br>mAb anti-alpha tubulin (1:250; 12G10 ,RRID:AB_1157911,Developmental Studies Hybridoma Bank), anti-GFP (1:250; #TP401, Torrey Pines), mAb anti-Ty (1:500;BB2, kindly provided by Dr. Lourido, Whitehead Institute), mAb anti-Myc (1:50; 9E10, Santa Cruz Biotechology), mAb anti-Myc Alexa488 conjugated (1:100;9B11, Cell Signaling Biotechnology), mAb anti-V5 (1:500; SV5-Pk1,BioRad), anti-beta tubulin (1:1000; kindly provided by Dr. Morrissette, University of California, Irvine), rat anti-IMC3 (1:2000), rabbit anti-IMC3 (1:2000; generated against the N-terminal 120 amino acids fused to His6), anti-human-Centrin2 (1:1000; kindly provided by Dr. Cheeseman, Whitehead Institute), anti-AAP4 (1:200), anti-ISP1 (1:500; kindley provided by Dr. Bradley, UCLA) .<br><br>Secondary Antibodies<br>goat anti-rabbit Oregon green(IFA 1:500, U-ExM 1:200; # O-11038, Invitrogen), goat anti-mouseALEXA594(IFA 1:500, U-ExM 1:200;#A11005, Invitrogen). Goat anti-mouseALEXA488 (#A11001), goat anti-mouseALEXA633 (#A21052), goat anti-ratALEXA488 (#A11006), goat anti-rabbitALEXA594 (#A11012), goat anti-guinea pigALEXA488 (#A11073), and goat anti-ginuea pigALEXA594 (#A11076) all used at 1:500 dilution and ordered from Invitrogen.
Validation	Primary antibodies were validated among others in the following studies:<br>anti-Ty: Bastin P, Bagherzadeh Z, Matthews KR, Gull K. (1996) A novel epitope tag system to study protein targeting and organelle biogenesis in Trypanosoma brucei. Mol Biochem Parasitol 77(2):235-9. PMID: 8813669<br><br>anti-beta tubulin: Morrissette NS, Sibley LD. (2002) Disruption of microtubules uncouples budding and nuclear division in Toxoplasma gondii. J Cell Sci. 115(Pt 5):1017-25. PMID: 11870220<br><br>rat anti-IMC3: Gubbels MJ, Wieffer M, Striepen B. (2004) Fluorescent protein tagging in Toxoplasma gondii: identification of a novel inner membrane complex component conserved among Apicomplexa. Mol Biochem Parasitol. 137(1):99-110. PMID: 15279956<br><br>anti-AAP4, rabbit anti-IMC3, anti-Myc (9E10) and anti-Myc-ALEXA488 (9B11) : Engelberg K, Chen CT, Bechtel T, Sánchez Guzmán V, Drozda AA, Chavan S, Weerapana E, Gubbels MJ. (2020) The apical annuli of Toxoplasma gondii are composed of coiled-coil and signalling proteins embedded in the inner membrane complex sutures. Cell Microbiol. 22(1):e13112 PMID: 31470470<br><br>anti-ISP1: Beck JR, Rodriguez-Fernandez IA, Leon JC de, Huynh M-H, Carruthers VB, Morrissette NS, Bradley PJ. (2010) A Novel Family of Toxoplasma IMC Proteins Displays a Hierarchical Organization and Functions in Coordinating Parasite Division. PLOS Pathog. 6:e1001094 PMID: 20844581<br><br>anti-V5 (SV5-Pk1): Rudlaff RM, Kraemer S, Streva VA, Dvorin JD. (2019) An essential contractile ring protein controls cell division in Plasmodium falciparum. Nat Commun. 10(1)2181 PMID:31097714<br><br>anti-human-Centrin2: Farrell M, Gubbels MJ. (2014) The Toxoplasma gondii kinetochore is required for centrosome association with the centrocone (spindle pole). Cell Microbiology. 16(1):78-94 PMID: 24015880<br><br>anti-alpha tubulin (12G10): reactive against T. gondii, validated for western blot and IFA according to providers website: [https://dshb.biology.uiowa.edu/12G10-anti-alpha-tubulin?quantity=1&product-form=2]<br><br>anti-GFP (TP401): validated for IFA and western blot in human cells, according to manufacturer's website: [https://www.labome.com/product/Torrey-Pines-Biolabs/TP401.html]

Eukaryotic cell lines

Policy information about cell lines

Cell line source(s)	human foreskin fibroblasts (HFF:ATCC #CRL-1634, BJ1-hTERT: RRID:CVCL_6573)<br><br>T. gondii RHΔKu80: Huynh MH, Carruthers VB (2009) Tagging of endogenous genes in a Toxoplasma gondii strain lacking Ku80. Eukaryot Cell. 8(4):530-9 PMID: 19218426<br><br>T. gondii TATiΔKu80: Sheiner L, Demerly JL, Poulsen N, Beatty WL, Lucas O, Behnke MS, White MW, Striepen B. (2011) A systematic screen to discover and analyze apicoplast proteins identifies a conserved and essential protein import factor. PLoS Pathog. 7(12):e1002392 PMID: 22144892<br><br>T. gondii TirIΔKu80: Brown KM, Long S, Sibley LD. (2017) Plasma Membrane Association by N-Acylation Governs PKG Function in Toxoplasma gondii. mBio. 8(3):e00375-17 PMID: 28465425
Authentication	T. gondii cell lines were authenticated for their particular biological function (e.g. high rate of homologous recombination in RHΔKu80 and functionality of Tir1 or Tet-transactivator in Tir1ΔKu80/TATiΔKu80 parasites). HFF cells were authenticated at the source and are periodically inspected by microscopy (observation of morphology).
Mycoplasma contamination	HFF/BJ1-hTERT and T. gondii parental lines are periodically tested for Mycoplasma contaminations, in which they tested negative. Established transgenic parasite lines have not been tested.

Commonly misidentified lines<br>(See ICLAC register)	None
